# What Do We Expect New Graduate Orthoptists to Do?

**DOI:** 10.22599/bioj.108

**Published:** 2018-04-24

**Authors:** Anna M. Horwood, on behalf of the Education Committee of British and Irish Orthoptic Society

**Affiliations:** 1 Infant Vision Laboratory, School of Psychology and Clinical, Language Sciences, University of Reading, Earley Gate, Reading Berks, RG6 6AL, GB

**Keywords:** orthoptics, curriculum, survey

## Abstract

**Aims:**

To validate the content of an updated orthoptic curriculum for the British & Irish Orthoptic Society (BIOS), BIOS members were surveyed about their views on what an orthoptist should be able to do soon after entering the profession.

**Methods:**

An online survey of all practicing members of BIOS was carried out. In 35 questions across 5 domains (professional behaviour, foundation knowledge and theory, investigation, management and research and literature skills) covering the range of orthoptic practice, orthoptists were asked about the breadth and depth of knowledge required. Results were analysed by the respondents’ working environment, experience, geographical region and teaching involvement.

**Results:**

325 orthoptists (27% of the membership) provided useable data, and 265 provided a full dataset. Orthoptists are frequently required to exercise considerable autonomy and responsibility for patient care from very early in their careers across many domains, often in the least-supervised environments. There was broad agreement across most core topics but wider variation in opinion in more peripheral domains. More experienced orthoptists value the wider medical aspects of orthoptic practice more highly.

**Conclusions:**

The survey confirmed that there is generally a good match between current undergraduate teaching and clinicians’ expectations of newly graduated orthoptists. It is clear that training must prepare graduates for a high level of professional autonomy from the earliest stages of their careers. There may be a place for targeting CPD provision for professionals at different stages in their careers.

## Introduction

Orthoptics is a small profession with close links between the small number of higher education institutions (HEIs) that offer orthoptics degrees and the profession as a whole. There has been no formal BIOS curriculum document since orthoptics became a graduate profession in 1992 because of an overall consensus about what core competencies were. Now, however, an increasing emphasis on orthoptists undertaking extended roles has made it clear that core competencies need to be more explicit and the boundary between core and extended roles needs to be more precisely defined in a formal document setting out the profession’s core priorities (British & Irish Orthoptic Society 2016).

All Allied Health Professions (AHPs) that are regulated by the Health and Care Professions Council (HCPC) work with regulators and bodies such as the Professional Standards Authority for Health & Social Care (PSAfHaSC), the Quality Assurance Agency (QAA) and the NHS Knowledge and Skills Framework (National Health Service 2016) to define overarching benchmarks for training and proficiency (Health and Care Professions Council 2013; Health and Care Professions Council 2009; Quality Assurance Agency 2001). Specifically, the HCPC sets out broad standards for all AHP education programmes, including requirements for admission to a course, programme management and resources, practice placements and assessment in order for new graduates to be admitted to the HCPC Register (Health & Care Professions Council 2009). Each profession’s HCPC Standards of Proficiency include generic skills and competencies common to all healthcare professionals, as well as topic-specific content arrived at by consultations with the professional bodies. In most cases, a profession’s curriculum document is used to further define this content. HEIs are free to decide how their courses meet these requirements. It is recognised that the HCPC Standards of Proficiency and Standards of Conduct, Performance and Ethics are at threshold level, and it is expected that pre-registration courses will teach beyond that baseline level.

The different UK AHPs have approached the preparation of their curriculum documents in many ways, and some have a wider remit than others (e.g. physiotherapy addresses competencies at all levels of the profession) (Chartered Society of Physiotherapists 2013). Most other professions started their curriculum revisions by referring to existing documents and used a working group approach involving relevant stakeholders. There were no directly equivalent published survey data available from the literature, although consultations within other professions may have resulted in internal reports. Because orthoptics is a small profession that has undergone many changes in workload in the past few years, and there was no existing curriculum document, consultation with the whole profession was both timely and possible. In the preparation of the curriculum framework, BIOS commissioned a large consultation with the membership to provide evidence of what is currently being expected of an Agenda for Change (AfC) Band 5 graduate HCPC Registered orthoptist (or Republic of Ireland equivalent). Band 5 is the UK entry level for qualified orthoptists, so Band 5 skills represent the core skill set for the profession. The curriculum framework is published by BIOS and is available online (British & Irish Orthoptic Society 2016). It details all the knowledge, skills and expertise BIOS expects orthoptists to possess as core skills (see Table 1). This paper outlines the main results and analysis of the survey.

**Table 1:** Questionnaire topics.

**Demographic Data**
Region (England North/Midlands/South, Wales, Scotland, Northern Ireland, Republic of Ireland)
NHS Grade banding (AfC band)
Type of practice (Community/Primary Care, Acute/Dist Gen. Hospital, Tertiary, Academic)
Roles undertaken (e.g. visual fields, stroke services, screening, botulinum clinics)
Years of practice
Whether they took undergraduate students on placement
**Professional behaviour (4 level ordinal scales)**
HCPC role, regulations, CPD (3 sub-topics)
Legal/ethical aspects of practice (e.g. data protection, confidentiality, safeguarding) (8 sub-topics)
Personal obligations (e.g. professional behaviour, reflective practice) (7 sub-topics)
Professional relationships, communication, team working with different groups (12 sub-topics)
Personal professional skills (e.g. equality and diversity, patient-centred care) (9 sub-topics)
Communication in specific situations (e.g. children, dementia, conflict resolution) (12 sub-topics)
Career, employee, employer issues (13 sub-topics)
**Foundation or Background Knowledge (4 level ordinal scales)**
General anatomy (3 sub-topics)
General physiology (7 sub-topics)
Embryology (2 sub-topics)
Child development and lifespan changes (5 sub-topics)
Pathology & general medical disease processes (15 sub-topics)
Detailed ocular anatomy and physiology (16 sub-topics)
Refractive and theoretical optics (10 sub-topics)
Principles and use of optical instruments and tests (11 sub-topics)
Psychology (6 sub-topics)
Core orthoptics theory (22 sub-topics)
**Investigation (6 level ordinal scales)**
Vision (9 sub-topics)
Ocular motility testing (17 sub-topics)
Binocular vision (8 sub-topics)
Suppression and correspondence (6 sub-topics)
Retinoscopy & refraction (11 sub-topics)
Ophthalmological tests (e.g. fields, slit lamp, OCT) (12 sub-topics)
**Management**
Management methods (e.g. prisms, occlusion, exercises) (26 sub-topics, 6 level ordinal scale)
Management of types of concomitant strabismus (19 sub-topics, 4 level ordinal scale)
Accommodation and convergence anomalies (9 sub-topics, 4 level ordinal scale)
Incomitant strabismus and nystagmus (18 sub-topics, 4 level ordinal scale)
Effects of general disease on ocular motility (10 sub-topics, 5 level ordinal scale)
Screening (8 sub-topics, 4 level ordinal scale)
“Extended roles” (e.g. glaucoma monitoring, low vision) (14sub-topics, 8 level scale)
Current use of ophthalmic drugs (before new legislation passed) (5 sub-topics, 3 level scale)
Ophthalmic procedures (e.g. botulinum, ophthalmic surgery) (19 sub-topics, 4 level scale)
**Research Skills**
Literature skills (9 sub-topics, 4 level scale)
Screening and audit outcomes (5 sub-topics, 6 level scale)
Research skills (e.g. reading a scientific paper, preparing grants) (22 sub-topics, 6 level scale)

## Method

The study was carried out to give BIOS an overview of what a newly qualified orthoptist was expected to be able to *do* at the time of the survey (February/March 2016) and, broadly, to what level of expertise across the range of different clinical environments.

An online questionnaire was developed and sent out to all the practicing members of BIOS. The author consulted the HEIs, HCPC documentation and the BIOS Education Committee during questionnaire development to arrive at five major domains of knowledge (Table 1). The survey incorporated detailed questions about orthoptic topics, as well as more generic skills common to all healthcare professionals required to register with the HCPC. Current course content from the three UK universities offering orthoptics degrees was used as reference. Data were also collected about the respondents’ years of experience, the scope of practice undertaken by the department in which they worked, geographical region and involvement with undergraduate education.

Respondents scored each topic on ordinal scales, which generally varied from “not important at all”, to basic/outline knowledge but no expectation of being practically competent, to being competent to deal with routine situations, to “being expert in the topic or autonomously fully responsible for care”. As this was a wide-ranging survey and covered both core orthoptics and more general medical education, the ordinal scales were in different levels of detail (between four and eight levels) depending on the topic. More scales points were used for specific orthoptic skills to establish a more detailed overview of opinions. This approach has allowed BIOS to collect fine-grain data on specific orthoptic topics, so as well as being used for the purposes of the curriculum project, it has also provided a valuable dataset and archive of current orthoptic practice for the profession. For some obviously core topics, such as the investigation of concomitant strabismus, the lowest point of the scale was “baseline ability to diagnose simple cases”; whereas, less core topics placed “not important at all” as the lowest level.

For the final published BIOS curriculum framework, the editor of the curriculum framework and the BIOS Education Committee then collated the responses, in some instances collapsed or refined topic headings, and assigned the topics to a common four level ordinal scale used across the final document (British & Irish Orthoptic Society 2016).

Data were analysed overall and also by the respondents’ AfC Band banding (or equivalent), working environment, years of experience, geographical region and whether they took students on clinical placement. Where appropriate, between-groups ANOVA and trend analysis was done where an ordinal scale of the sub-groups was applicable (e.g. AfC Band or years of experience). This assumes that AfC banding and years of experience represent broadly linear scales. When these ordinal scales were analysed, the responses of orthoptists working on academic pay scales were excluded as their clinical equivalent banding was less likely to reflect their place in the conventional clinical hierarchy, and a few academics do not work clinically.

For the majority of data, there were no differences between the opinions of those on different banding or working in different environments, regions, or with longer or shorter experience. There was an overall consensus on what skills and knowledge were necessary, so only notable findings from the sub-analyses are reported.

## Results

There were 325 respondents who filled in part or all of the survey, with 265 completing all 43 questions with the 378 sub topics. This represents the views of 27% of the 1194 practicing BIOS membership on the date of circulation.

The regional representation was broadly in proportion to the membership in each region, as were the responses from different types of practice (e.g. primary, secondary, tertiary care or academic.

Fifty-five percent of the respondents were AfC Band 7 or above (i.e., with supervisory, specialist or managerial responsibility), while only 6% were in Band 5 roles. Therefore, the majority of the responses reflect what a more senior orthoptist expects of their junior colleagues, rather than the current experience of those individuals. Forty-eight percent of respondents had been in practice for over 20 years. Eighty percent of respondents worked in places where undergraduates are taken on clinical placement.

Most departments engaged in many roles beyond pure hospital orthoptic practice (e.g. screening, glaucoma work, stroke services, work in special schools). With the exception of four academic respondents and two respondents from a tertiary referral centre, all worked in units where other roles were undertaken. The most common were visual field services (74%), school entry screening (66%), special schools/special educational needs (68%), low vision services (37%), stroke services (89%), specific literacy difficulties (44%), glaucoma services (40%) and use of drugs under patient group directives (46%).

### Overall Analysis

#### Professional behaviour

Most respondents expected a level of expertise to at least everyday competence and mandatory training, but 30% to 40% of respondents expected ability beyond this level and would expect new graduates to deal with communication with all patients and colleagues, adherence to best practice guidelines and adherence to legal and ethical frameworks at the highest levels.

#### Foundation/background knowledge

It was clear that a comprehensive knowledge of general medical issues was required (e.g. general anatomy and physiology, disease processes, development and ageing, the role of other professions and psychology) to enable orthoptists to at least be able to understand any implications for their patients and to understand any letters and reports in hospital notes. As topics approached core orthoptics (e.g. ocular anatomy and physiology, optics, vision and binocular vision science) or conditions that directly affect orthoptic patients (myasthenia, thyroid eye disease), the highest (expert) level of knowledge was expected. There was a wide range of opinions concerning child development and psychology; views varied between only needing to know an outline and knowing precise detail of how they affected orthoptic practice.

#### Investigation methods

Orthoptists were generally expected to be able to choose, use and interpret tests independently for a typical orthoptic caseload and only seek advice for complex cases. They also were expected to possess a high level of knowledge about the indications and interpretation of tests generally performed by others (e.g. optometric and ophthalmological testing) but would not necessarily be expected to be expert in their use themselves. There was some mismatch between a high level of competence in skills already being taught and assessed by the universities (e.g. the use of a slit lamp and retinoscopy) and the expectations of clinicians, who would often not expect new graduates to use them at all in a first job.

#### Management methods

**Figure 1:**
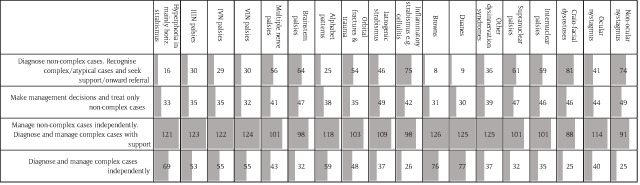
Number of responses to the question on autonomy in decision-making in incomitant and complex ocular motility diagnoses. Grey bars represent relative proportion of responses in each column.

Orthoptists were generally expected to manage their own caseload independently from the start, without direct supervision, and only seek advice and support in complex cases. There was more variability in opinions for complex incomitant strabismus, where 10% to 25% of respondents would only give the simplest of such cases to new graduates. Although they might expect them to diagnose them, they would not expect competence in management. However, a slightly larger proportion (15% to 25%) would expect the same patients to be managed entirely independently (Figure 1).

**Figure 2:**
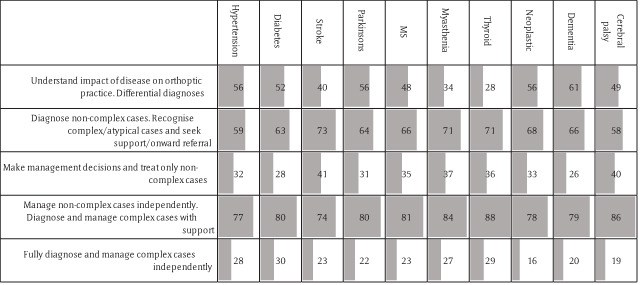
Number of responses to the question on the orthoptist role in general medical conditions that impact adult orthoptic practice. Grey bars represent proportion of responses in each column. Note wide variation of opinion.

There was an even wider distribution of views on the management of patients with complex general conditions, such as stroke, MS and Parkinson’s disease (Figure 2). While 35% would expect a Band 5 orthoptist to only initially diagnose the orthoptic aspects of the case and seek further advice, around 25% would expect them to manage all orthoptic aspects of the cases independently and only seek support when required. Forty-eight percent of respondents would expect an early career orthoptist to be able to carry out primary vision screening.

By definition, a Band 5 orthoptist would not be expected to be able to take on extended roles and would only be expected to know the broad outlines of how orthoptists exercise these roles. They might be expected to carry out a test used *within* extended role (e.g. visual fields at the direction of others in a glaucoma service) but with minimal responsibility. The definition of an extended role appeared to involve either taking independent diagnostic or management decisions in conditions beyond the core topics of strabismus and binocular vision, developing or managing a service or working as a core member of a multidisciplinary team where additional skills or knowledge were required.

#### Research and literature skills

This was another area where there was a mismatch between current training and expectations. Undergraduates are currently expected to demonstrate expertise in literature analysis and appraisal, and most are expected to be able to carry out an original personal research project. However, these skills are currently not seen as the preserve of early career clinicians, where only a very basic understanding was expected, even in the context of local audit.

### Analysis By Band

**Figure 3:**
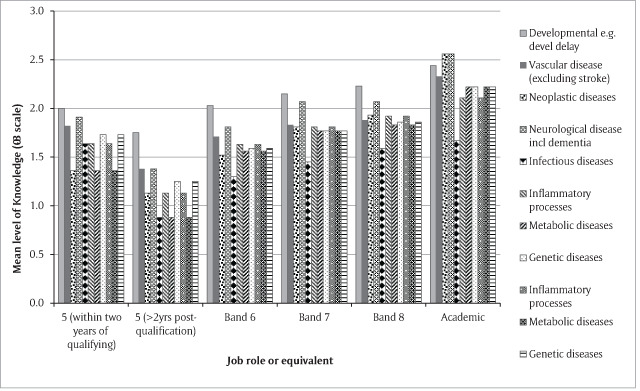
Opinions of the importance of knowledge of general pathology analysed by job role. Y-axis indicates mean level of knowledge required on a four point scale where 0 = not useful at all; 1 = know the outline; 2 = understand terms in letters/reports and orthoptic risk factors if abnormal; 3 = know detail of how abnormalities affect orthoptic practice.

The most notable finding was that beyond the immediate post-graduation period, where opinions are likely to have been driven by academic priorities as a student, rather than personal experience, the more junior the respondent, the less they valued the medical aspects of orthoptics (e.g. knowledge of general anatomy and physiology [linear trend F(df1,251) = 32.62, p < 0.001], pathology and disease processes [linear trend F(df1,271) = 32.62, p < 0.001 (Figure 3)], embryology [linear trend F(1,264) = 26.06 p =< 0.001], child development [linear trend F(1,269) = 11.40, p = 0.001]).

In contrast, they put a higher value on core orthoptic skills in the management of concomitant strabismus (linear trend F(1,199) = 7.93 p = 0.005) and the effects of disease on ocular movements than did their more experienced colleagues (linear trend F(1,198) = 9.94 p = 0.002).

Newly qualified respondents were much less concerned about having knowledge of the extended roles of the orthoptist, but within two years, they had overtaken their Band 6 and Band 7 colleagues to consider them as important as their Band 8 managers.

In general, and not unexpectedly, the academics involved in university teaching had higher expectations overall than pure clinicians, and particularly for some aspects of practice (e.g. retinoscopy and refraction [p < 0.002 in all comparisons between academics and clinicians of Band 6 and above]). While there was strong agreement between academic orthoptists and their clinical colleagues on most orthoptic topics, there was a large difference in their rating of research, audit and literature skills. Most clinical orthoptists only consider a very basic understanding of research to be necessary, although most new graduates would be expected to carry out a simple audit with some support. The most newly qualified also valued these skills, but this high rating for research dropped away by two years after qualification.

### Assessment By Years in Practice

As with grade banding above, the more experienced orthoptists valued knowledge of general medicine more than less experienced staff (F(1,247) = 12.01, p = 0.001). There was a gradual decline in estimates of value of professional relationships (linear trend F(1,226) = 4.128, p = 0.043). The most newly qualified respondents felt they were expected to manage strabismus to a higher level than did their more experienced colleagues (significant linear trends in all types of strabismus), particularly in the case of incomitant strabismus (linear trend F(1,198) = 10.35, p = 0.002). They also felt research skills were more important.

### Analysis By Working Environment

Many respondents worked in multiple environments (e.g. primary and secondary, or academic and tertiary), so statistical analysis would have been too complex and underpowered to be meaningful. Overall impressions were that tertiary referral centres appeared to value professional behaviour, personal obligations and communication skills more than more general clinics. In contrast, they did not expect such high level patient management autonomy in junior staff. This is likely to reflect a more complex caseload, where junior staff might need more support. Tertiary centres had higher expectations of awareness of extended roles across the whole range of topics and also greater use of research skills.

### Analysis By Region

This analysis was limited by small numbers in the non-English regions surveyed, but it does reflect a tendency for higher expectations from orthoptists not working in England. This was particularly noticeable for Northern Ireland and the Republic of Ireland (but there were only 11 and 13 respondents respectively and statistical significance was not reached [p > 0.05] in any comparison).

### Assessment By Involvement with Students

Overall, people working in departments that took students had lower expectations of new graduates, particularly in terms of research and literature skills (p < 0.008 in all categories), extended roles (t(241) = 71.63, p = 0.004) and professional behaviour (t(279) = 2.01, p = 0.039) than those who did not have contact with students. In the case of research and literature skills and extended roles, this could reflect a more realistic perception of what students actually demonstrate on placement, but in part could also reflect personal limitations of the supervisors. For example, if a supervisor lacks confidence or experience of research or an extended role, they may avoid questioning a student in depth to explore their knowledge. The only exception was in the management of concomitant strabismus, where those who took students appeared overall to expect slightly more of their new staff than those who did not.

## Discussion

This survey of orthoptists achieved the overall objectives of BIOS in providing a snapshot of current practice and the expectations of what early-career orthoptists in the UK and Ireland need to do. There were no major surprises in the overall responses, but some interesting findings may help BIOS and the universities adjust expectations and plan for future development.

A response rate of 27% is high for any survey, especially one which took so long to fill in. The survey took at least 45 minutes to complete, so it is unsurprising that some failed to complete the whole form, but as questions were analysed separately and all completed the first few questions, which allowed their responses to be categorized by band, environment et cetera, their partial data were still useful. Overall, the respondents were representative of the profession as a whole. The survey was only sent to BIOS members, not all HCPC registrants, but it was commissioned by BIOS, not the HCPC, to represent members’ priorities. If all HCPC registrants had been surveyed, many non-practising orthoptists (who maintain registration but do not maintain BIOS membership) might have made the survey less representative of current practice.

There was a somewhat higher proportion of senior orthoptist respondents than represented in overall BIOS registration. Most of the respondents were in more senior roles than the band they had been asked to describe, but as they are often the ones who direct more junior staff, their views are just as useful. The small number of Band 5 responses is mainly a reflection of the small number of such posts nationally, because some new graduates enter the profession at a more senior level or quickly move up from Band 5. It therefore highlights how much responsibility and expertise is required within the profession soon after qualification.

The vast majority of respondents agreed on the core orthoptic skills. These are skills on which orthoptics focuses, where orthoptists deal with new cases who have been referred from primary care services because of a need for a more expert opinion and where they would be expected to act autonomously without direct supervision, even from another orthoptist. Very high levels of autonomy, personal responsibility for patient care and reflective practice were expected from the start. Wider variation of opinion was often found in more peripheral topics. Overall, a very wide spread of general medical knowledge is necessary for modern practice, reflecting how orthoptists are embedded in hospital environments, dealing with patients with multiple morbidities, the full range of general ophthalmological conditions and interacting with many other health professionals as part of their core, not extended, practice. The orthoptist is often the first eye professional a patient sees as a new case in an eye clinic, so they must be able to recognise and prioritise risk and make appropriate onward referral for ophthalmological and general medical conditions if necessary. Orthoptists are nearly always expected to work independently from the time of registration and seek support only when they consider it necessary. A formal preceptorship period is recommended for all newly qualified staff, but this is not available in all areas, and often there are less formal support arrangements.

Regions where recruitment has traditionally been challenging and which has been an issue for the profession for some years (personal communication with BIOS officers), where services are stretched and where orthoptists may work single handed (particularly Ireland and rural areas) expect more initial responsibility from their staff. Close support for their new graduates may be impossible in practice. It may be an area of concern for the profession that the most able graduates may be more likely to apply for (or be offered) the “best” jobs in high profile tertiary centres, where they get the most support from preceptorships, while those perhaps in need of more support at first may actually be working in areas where it is most difficult to achieve.

It was evident that some tertiary centres would not allow new staff to see or independently manage complex cases, while smaller district or community services would expect them to manage everything and only seek advice when necessary. This may reflect a more complex case load in tertiary centres, but it does mean that the universities have to continue to teach to a very high level so that any new graduate would be able to deliver safe patient care in the more common smaller acute trust roles.

It is somewhat of a paradox that tertiary referral centres often attract the most able applicants for posts, but in practice, if they are then only expected to see simpler cases, it may mean that the orthoptists who are arguably the most able to deal with complex cases are the least likely to be getting experience with them at first. Conversely, their cohort fellows, who may not have even been shortlisted for the “better” jobs due to a lesser classification degree, might end up being given the most responsibility on qualification. If both then wish to move to another post, the less able candidate may actually be able to claim wider experience to put on a CV. This survey highlights that both tertiary centres, which limit some types of clinical experience to new graduates, and those smaller departments allowing them to see everything should be aware that neither situation is ideal. Tailored preceptorship, mentorship, structured CPD and training across the whole curriculum are vital for all.

There were two areas where there appeared to be a mismatch between what is being taught at the universities and what is expected of early career orthoptists: research and some non-orthoptic but still core investigation methods. Personal communication with all three universities offering orthoptics degrees confirmed that undergraduates are taught and assessed in retinoscopy, refraction and many ophthalmological examination techniques and all have some research methods experience. So most have good skills as they qualify, but they often do not appear to be expected to use them in their first job because many respondents appeared to consider them to be part of extended roles, which may be used as a basis for promotion. The risk of the situation is that research skills and ophthalmological techniques taught to a relatively high level to undergraduates are then lost due to disuse. They then may have to be relearned later but often may not be.

Clinicians seem to regard research and analysis activity as something “other more senior people do” despite lower level audit requiring identical skills. HCPC Standards of Proficiency Section 12 (Health and Care Professions Council 2013) makes it clear that research and audit skills are vital for all registrants. More recently, the Council for AHP Research has produced a Position Statement placing use of research skills as core too all AHP practice and education (Council for Allied Health Profession Research 2016).

There may be a perception that by asking junior staff to maintain some general ophthalmological skills (e.g. slit lamp) at an entry level it may hinder promotion chances, which may be based on taking on new extended roles later. The counterargument to this is that clinical skills themselves are rarely the argument for promotion; it is the decision making, autonomy, research, audit or management roles in a situation that often determines grade progression. The definition of an extended role appears to involve either taking independent diagnostic or management decisions in conditions beyond the core topics of strabismus and binocular vision (usually in roles formerly performed by medical staff, such as glaucoma or AMD), developing or managing an independent service (e.g. special schools), or working as a key decision-maker in a multidisciplinary team where additional non-orthoptic skills or knowledge were required (e.g. dyslexia/SLD or stroke). The use of core skills, such as investigating a stroke patient, being a minor part of a collaborative research group or being able to use a slit lamp, are not extended roles.

In both the above situations, there appears to be a self-perpetuating barrier for junior staff as their middle-grade colleagues may not encourage them to develop or maintain skills taught to a competent level at university, despite leaders of the profession encouraging research and the development of extended roles. There may be a role for targeting CPD in research for middle grade clinicians in order to raise its profile across the whole profession: supporting these orthoptists to maintain or reinforce their own skills not only gives them a platform for their own further personal development, but also may give them confidence to encourage new graduates to engage in research and audit too. The profession has a great need to maintain a pool of research skills so that orthoptists are ready to undertake clinical research themselves and not wait for any research to be driven or undertaken by other professionals.

There were few notable differences when the data were broken down by the different groups, but it was interesting that the general medical aspects of orthoptics were valued more highly by more senior staff. This is likely because they see the bigger picture, while younger orthoptists are still concentrating on orthoptics itself. Band 5 orthoptists did not value psychology, child development or general medicine/pathology very highly. These topics are generally taught early in the undergraduate courses, and it seems to take years of experience to see how important a broad general medical background is to delivering the best patient care. The profession as a whole should be aware that new graduates qualify with relatively high level general medical knowledge because they will have been formally examined in it, but then some more junior orthoptists seem to feel these topics become lower priority in practice.

Although not statistically significant, there seemed to be a tendency for newly qualified orthoptists (Band 5 with less than two years’ experience) to reflect the high standards of their teachers who also reflect the views of senior members of the profession. It appears that junior orthoptists then go through a phase of thinking that higher level skills are less relevant, but then as they climb the career ladder, they rediscover them. This probably means that people working long-term in Band 5 and 6 roles are in need of most CPD to maintain high standards. Early career CPD could perhaps be better targeted to the specific skills and knowledge that seem to be lost or given low priority. Universities could share with students about to graduate how their priorities, views and developing practice may change after qualification and to beware that they may unwittingly neglect topics they subsequently find they have to relearn.

The Band 5 respondents felt that they were expected to manage all types of strabismus (especially incomitant strabismus) to a higher level than their more experienced colleagues actually expected them to. This could represent a risk if the junior orthoptist feels they should be able to deal with a case independently and so do not ask for help. Mentoring and preceptorship could be directed to mutual awareness of this risk.

It is difficult to interpret the statistics about professional values and behaviour, which seemed to be less valued by more experienced and older orthoptists. It may be that they genuinely value these skills less, or it may just reflect that they are so embedded in their behaviour that they are too obvious to be voiced. Alternatively, more recent emphasis within the NHS on making such values and behaviour explicit rather than implicit may have passed by experienced staff who may have been working for years in the same role.

Orthoptists involved in the training of undergraduates generally had lower expectations of new graduates than those who did not. This probably represents a more realistic viewpoint but might serve to alert orthoptists in departments who do not take students that new graduates might not be quite as skilled on qualification as they expect.

This project was undertaken to address a particular urgent professional need, rather than as a stand-alone research project. An alternative, more step-by-step method might have been to adopt a Delphi approach (Iqbal and Pipon-Young 2009) to identify important themes and their relative priorities, but this would have been a complex and time consuming process in view of the many topics covered. Further research stemming from this project could focus on qualitative research, such as content analysis from specific focus groups to explore professional attitudes.

These data offer a snapshot of current orthoptic practice, so a repeat of the survey in a few years might allow BIOS to monitor professional changes over time.

## Conclusion

There was overall agreement across the profession about what new graduates should be able to do, especially for core clinical orthoptic skills. The overwhelming impression from the survey was that newly qualified orthoptists are often expected to work to very high standards of autonomy and professional practice from the start. They need not only specific orthoptic expertise, but also a wide general medical education. Large departments that can expect to provide close supervision or structured training pathways and rotations as may be offered by other professions (e.g. physiotherapy) are unusual. Undergraduate training must therefore prepare all graduates for largely independent practice, because some must start their career ready to work largely autonomously. The profession might consider targeting different types of CPD at different career grades.
